# Eutrophication triggered changes in network structure and fluxes of the Comacchio Lagoon (Italy)

**DOI:** 10.1371/journal.pone.0313416

**Published:** 2025-01-08

**Authors:** Katalin Patonai, Mattia Lanzoni, Giuseppe Castaldelli, Ferenc Jordán, Anna Gavioli

**Affiliations:** 1 Department of Environmental and Prevention Sciences, University of Ferrara, Ferrara, Italy; 2 Département de Sciences Biologiques, Université de Montréal, Montréal, Canada; 3 Department of Chemistry, Life Sciences and Environmental Sustainability, University of Parma, Parma, Italy; 4 Institute of Biological Research (NIRDBS), Cluj-Napoca, Romania; National Research Foundation - South African Institute for Aquatic Biodiversity, SOUTH AFRICA

## Abstract

Coastal lagoons, which cover about 13% of coastline, are among the most productive ecosystems worldwide. However, they are subject to significant stressors, both natural and anthropogenic, which can alter ecosystem services and functioning and food web structure. In the Comacchio Lagoon (Northern Italy), eutrophication, among other minor factors, transformed the ecosystem in the early 1980s. Here, we compiled available data for the lagoon into trophic networks (pre- and post-transformation), analyzed the ecosystem using local and global network analysis, and computed trophic fluxes of the two periods. For comparability, the networks of two periods (i.e., pre- and post- transformation) were aggregated into food webs with 23 nodes. We found differences in the trophic networks before and after eutrophication, resulting in some decrease in complexity, increase of flow diversity, and an overall shortening of the food chain. A crucial aspect of this change is the disappearance of submerged vegetation in the lagoon and the increased importance of cyanobacteria in the post-eutrophication period. We provide an approach to better understand ecosystem changes after severe disturbances which can be extended to biodiversity conservation and for the management of coastal resources in general.

## Introduction

Coastal brackish lagoons cover about 13% of coastlines globally [1] and are one of the most productive habitats worldwide due to the interactions between marine and inland waters [2]. These shallow, semi-enclosed systems provide numerous ecosystem functions, including nursing grounds for juvenile fish and stopover and feeding sites for migrating birds, especially at mid-latitudes [3]. However, they are subject to human pressures which alter the diversity and functioning of aquatic ecosystems. Pollution, habitat loss and alteration, biotic homogenization due to the spread of invasive species and climate change are some of the significant impacts threatening these aquatic ecosystems [4–6].

Stressors—natural or anthropogenic—may result in changes in ecosystem services and functioning and community-level effects evident in food web structure [7–9]. Eutrophication for instance can overload the system with nutrients creating oxygen depleted dead zones (e.g., in the Gulf of Mexico), which necessarily reduces the length of the food chain [10] and can shift the trophic position of invertebrates [11]. Habitat loss (e.g., loss of seagrass beds) or habitat alteration (e.g., increased sedimentation) are also major forces driving structural change by reducing primary production with cascading effects to higher trophic levels [12]. Biotic impacts, such as the spread of invasive species, can also result in community-wide changes affecting food web topology [8, 13]. Depending on the spatial or temporal extent of the impact on the actual habitat, the system-level consequences can be temporary or long-term [14]. Stressors usually occur concurrently, especially in coastal ecosystems where human activity is present. In recent years, increased attention has been given to the restoration of coastal lagoons, as their health is closely linked to the provision of ecosystem services for human societies [15]. However, these efforts are often site-specific and require ìn enormeus amount of resources. In these scenarios where resources are limited, it is essential to maintain an ongoing monitoring effort to support effective management actions.

While ecological studies focusing on individual species remain valuable, they are insufficient to account to the ecological and socio-economical complexity of the coastal lagoons ecosystems. Consequently, over the past two decades, there has been a marked increase in attention for modelling studies, concerning coastal shelf food webs. These models, relying on both qualitative (conceptual) or quantitative analyses, required comprehensive data collection encompassing a wide range of species [16]. A deeper understanding of community structure and function can be gained through a system-level approach from ecological networks and trophic modelling [16–19]. Trophic models, for example, can address ecosystem features such as thermal stress, life cycling, or regime shift [7, 20, 21]. Furthermore, standardized methodology (e.g., Ecopath with Ecosim models, [22]) are key to be able to compare multiple systems and reveal ecological patterns and processes among them [23, 24]. At the same time, data limitations (especially the lack of quantitative information) can hinder research efforts [16, 25] as for example with under-studied taxonomic groups (e.g., benthic invertebrates).

Among knowledge gaps, also certain drivers of food webs changes are insufficiently addressed such as species interactions, climate change, habitat alterations compared to others more discussed such as the effect of fisheries [16]. Despite these challenges, examining networks in a holistic way has several advantages: it can point out knowledge gaps [26] and identify important roles of functional groups [27–29]. Therefore, where data are limiting, simple connectance food webs relying on binary (presence/absence) trophic information can be the starting point for these system-level studies, which can be followed with more complicated and quantitative models (e.g. weighted trophic networks).

This study focused on the Comacchio Lagoon, in the north-western Adriatic coast of Italy. Here, in the mid 1980s, fishing industry and human activities caused profound transformations in the ecosystem and in the food web [30, 31]. After a major eutrophication event in the 1980s, an extensive survey revealed a notable shift in the lagoon ecosystem [30]. Prior to the transformation (i.e., pre-transformation period, PRE), the lagoon featured usual representative functional groups, such as aquatic vegetation, zooplankton, rotifera, benthic community and fish. However, following the eutrophication event (i.e., post-transformation period, POST), the lagoon has transformed into a cyanobacteria-dominated system with a food web that is nearly completely missing previously abundant organisms such as bottom vegetation and benthic fauna [30]. As a result, the Comacchio Lagoon provides an ideal setting to study changes in food webs at the ecosystem level. This is particularly relevant giving its importance for biodiversity conservation and the management of coastal resources, also considering the limited availability of comprenhensive recent data on food web in the lagoon.

Given the fragmented nature of the available data and the lack of comprehensive trophic networks, this study aims to *i)* construct trophic networks for the Comacchio Lagoon system during both the pre- and post-transformation periods. Furthermore *ii)*, this study provides the first comparison of the temporal differences in the food webs (pre- and post-transformation periods) using network analysis, with the provision of *iii)* energy fluxes within each network.

Our findings offer valueable insights into species interactions and changes in community structure following a significant disturbance. Furthermore, by identifying key functional groups and altered trophic pathways, our results provide information for ecosysten management.

## Methods

### The Comacchio Lagoon ecosystem

**Fig 1:**
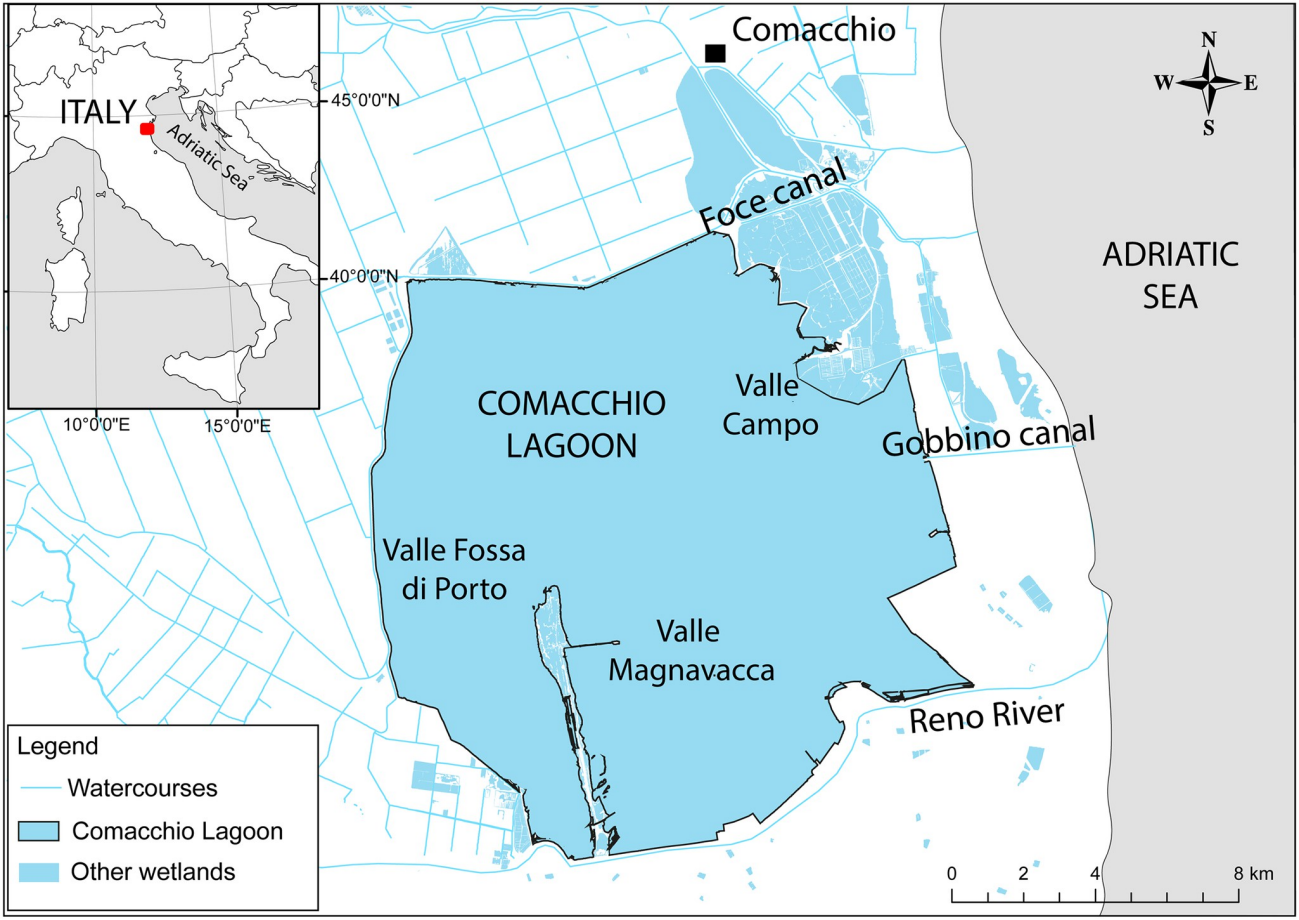
Map of the Comacchio Lagoon.

The Comacchio Lagoon is a semi-enclosed coastal body of water in the northeastern part of the Adriatic Sea (44°37′12.6′′N, 12°09′35.2′′E), in northern Italy. At this time, the lagoon comprises of three main basins (Valle Campo, Valle Magnavacca, and Valle Fossa di Porto) for a total extension of ~100 km^2^. The Comacchio Lagoon is connected to the Adriatic Sea by two channels whose opening is human regulated: the Foce and Gobbino canals. In the southern part of the lagoon, the Reno River contributes freshwater to the lagoon, this water diversion is artificially regulated (Fig 1). Annual salinities of the lagoon range from 16 to 36 ppt, with an annual average value of 28 ppt. Water depth varies from a few centimetres on the shallowest sandbanks to a maximum of 2.5 meters in the deepest channels with an average depth of ~ 1 m. The substrate is mainly constituted by mud, but sand and harder substrates (e.g., wooden navigation piles) are also present. The study area has a Mediterranean climate with winter temperatures below 0°C and summer temperatures above 30°C. In recent years, water temperature of Valli di Comacchio ranged from 3°C in winter season to more than 30°C in summer. The annual rainfall is between 600–700 mm, with the highest amount in autumn.

In spite of the traditional use of the Comacchio Lagoon for fishing, which began a long time ago, the lagoon underwent a complete physical and ecological transformation in the 1980s, due to a severe eutrophication event that altered the food webs [30, 31]. For instance, chlorophyll-a, which is considered an indicator of coastal primary production due to its foundational role in the pelagic food web and its direct and indirect effects on benthic primary production, was nearly absent during the pre-transformation period, whereas higher concentration was observed in the post-transformation period (Table 1).

**Table 1:** Environmental data for the pre-transformation (PRE) and post-transformation (POST) periods. For each variable, the year of measurement, mean values, reference and percentage change are shown. The percentage change is calculated as (x_post_−x_pre_)/x_pre_×100.

Environmental variable (unit)		PRE		POST		PercentChange (%)	
	Year	Value	References	Year	Value	References	Value
Mean water depth (m)	1971	1.10	Colombo et al., 1988 [75]	2011	1.20	Bernardi et al., 2012 [77]	9.09
Mean annual water temperature (°C)	1971	15.33	Ceccherelli & Ferrari, 1976 [76]	2009	19.28	Lanzoni et al., 2022 [78]	25.77
Mean summer water temperature (°C)	1971	24.16	Ceccherelli & Ferrari, 1976 [76]	2009	25.69	Lanzoni et al., 2022 [78]	6.33
Mean annual water salinity (ppt)	1971	35.19	Ceccherelli & Ferrari, 1976 [76]	2009	36.10	Lanzoni et al., 2022 [78]	2.59
Mean summer water salinity (ppt)	1971	35.92	Ceccherelli & Ferrari, 1976 [76]	2009	37.00	Lanzoni et al., 2022 [78]	3.01
Mean annual Chlorophyll-*a* (μg/L)	1978	<1.00	S.I.VAL.CO, 1980 [49]	2009	54.00	Lanzoni et al., 2022 [78]	5300.0

### Data

A literature survey on Web of Science (WoS) was conducted to identify available biomass data for the Comacchio Lagoon in the pre-transformation and post-transformation periods (i.e., prior and post the 1980s). The search on WoS was conducted on December 11, 2023 using the search string”Comacchio AND biomass*” in title, abstract and keywords of the manuscript. To derive specific information on biomass data, we decided to not include other synonyms in the query. The search produced 14 publications, from which two had information on Comacchio [31, 32].

Therefore, it was not possible to obtain data for the Comacchio Lagoon food web using only white literature. Consequently, the research was complemented using gray literature and survey data for fish (S1 Table). Species reported in the literature without biomass information were included in all analyses except for the calculation of energy fluxes within the food web, which requires biomass data. Taxonomic nomenclature for species mentioned in the original sources has been revised based on the most recent accepted names available in the World Register of Marine Species (WoRMS [33]).

To identify predator-prey relationships within the food web (S2 Table), information on food items of each taxa was obtained from FishBase [34], SeaLifeBase [35] and WoRMS [33] and references in S1 Table.

All the data has been classified as representative of the pre-transformation period when the records were kept from 1970 to 1980. All available data collected from 1996 onwards has been classified as representative of the post-transformation period (Table 2, S1 Table). Environmental data were also collected from literature (Table 1) and the direction and percent of change (%) was calculated using (x_post_−x_pre_)/x_pre_×100 formula.

**Table 2:** All available information of taxa identified from literature in the Comacchio Lagoon during the pre-transformation (PRE) and post-transformation (POST) periods. For each taxon, the presence during the PRE and POST periods is indicated and its functional group.

Taxa	Functional Group	PRE	POST
*Alitta succinea*	Surface Deposit Feeders	x	
Bivalvia larvae	Suspension Feeders	x	
*Chironomus salinarius*	Subsurface Deposit Feeders	x	
*Dicentrarchus labrax*	Dicentrarchus labrax	x	
*Haminoea hydatis*	Surface Deposit Feeders	x	
*Hydrobia* sp.	Surface Deposit Feeders	x	
*Idotea balthica*	Scrapers	x	
*Lamprothamnium papulosum*	*Lamprothamnium papulosum*	x	
*Littorina* sp.	Grazers	x	
*Maja squinado*	*Maja squinado*	x	
*Palaemon elegans*	Shrimp	x	
*Platichthys flesus*	Flatfish	x	
Polychaeta larvae	Surface Deposit Feeders	x	
Polychaeta mobile	Benthic Predators	x	
*Ruppia cirrhosa*	*Ruppia cirrhosa*	x	
Small invertebrate larvae	Filter Feeders	x	
*Solea solea*	Flatfish	x	
*Tritia neritea*	Grazers	x	
*Upogebia pusilla*	*Upogebia pusilla*	x	
*Acartia*	Surface Deposit Feeders		x
*Ardea alba*	Birds		x
*Ardea cinerea*	Birds		x
Balanidae sp.	Suspension Feeders		x
Chironomidae	Subsurface Deposit Feeders		x
Ciliata	Surface Deposit Feeders		x
*Crangon crangon*	Shrimp		x
Cumacea sp.	Grazers		x
Diatom	Diatom		x
Dinoflagellata	Dinoflagellata		x
*Egretta garzetta*	Birds		x
*Engraulis encrasicolus*	Engraulis encrasicolus		x
Hydrobiidae	Surface Deposit Feeders		x
Malacostraca	Grazers		x
Mysida	Grazers		x
Nanocyanobacteria	Nanocyanobacteria		x
Nemertea	Benthic Predators		x
Nereididae sp.	Surface Deposit Feeders		x
*Palaemon* sp.	Shrimp		x
*Phalacrocorax carbo*	Birds		x
*Phalacrocorax pygmaeus*	Birds		x
Picocyanobacteria	Picocyanobacteria		x
*Podiceps cristatus*	Birds		x
*Podiceps nigricollis*	Birds		x
Polychaeta	Surface Deposit Feeders		x
Shrimp	Shrimp		x
Spionidae	Benthic Predators		x
*Sterna hirundo*	Birds		x
*Sterna sandvicensis*	Birds		x
Syllidae sp.	Surface Deposit Feeders		x
Tanaidacea sp.	Grazers		x
Turbellaria	Benthic Predators		x
Zoea larvae	Grazers		x
Actiniaria	Actiniaria	x	x
Amphipoda	Scrapers	x	x
*Anguilla anguilla*	*Anguilla anguilla*	x	x
*Anguilla anguilla* Juvenile	*Anguilla anguilla*	x	x
*Aphanius fasciatus*	*Aphanius fasciatus*	x	x
*Atherina boyeri*	*Atherina boyeri*	x	x
Bivalvia	Suspension Feeders	x	x
*Carcinus aestuarii*	*Carcinus aestuarii*	x	x
*Chelon auratus*	Mugil spp.	x	x
*Chelon ramada*	Mugil spp.	x	x
*Chelon saliens*	Mugil spp.	x	x
Copepoda	Copepoda	x	x
Crustacea	Scrapers	x	x
Detritus	Detritus	x	x
*Gammarus* sp.	Scrapers	x	x
Gastropoda	Grazers	x	x
Gobidae	Gobidae	x	x
*Monocorophium insidiosum*	Surface Deposit Feeders	x	x
*Mugil cephalus*	Mugil spp.	x	x
Oligochaeta	Subsurface Deposit Feeders	x	x
Polychaeta sedentary	Surface Deposit Feeders	x	x
Rotifera	Filter Feeders	x	x

### Food web construction and network analysis

Food web construction and network analysis were based on literature information from FishBase [34], SeaLifeBase [35] and WoRMS [33]. Detailed predator-prey relationships were not uniformly expressed using weight values (i.e., biomasses). Therefore, to include all information, binary (unweighted) connectance networks were compiled with predator-prey relationship for the pre-transformation and for the post-transformation periods, respectively (S2 Table).

The food webs were connected (single-component) networks, which were symmetrized (by taking the sum of the links between each node, x_*ij*_ + x_*ji*_). The two networks (pre- and post-transformation) differed in resolution, especially for invertebrates. To make the networks more comparable, we aggregated invertebrates based on functional groups (FG) [36]. Also, the four mullet species were combined as *Mugil* spp. and all water bird species were merged into one node called *Birds* (Table 2). This low-resolution (i.e., highly aggregated) approach provides less detailed information on the trophic system but increases comparability (while finer resolution increases noise and decreases comparability). Food webs were visualized in R software [37] using the igraph package [38].

To describe each network overall, six global indices were computed: the number of nodes (N), the number of links (L), network density (D), weighted overall graph clustering coefficient (CL), average distance (d), and small-world index (SW). The number of nodes (N) provides the number of species or functional groups in the food webs (node names were taken exactly as they were described in the original sources, Table 2). The number of links (L) describes the binary (0/1) trophic (feeding) connections between the nodes. Network density (D) is the connectance, describing the number of actual (realized) links (i.e., predator-prey relationships) from all mathematically possible links, computed as D = 2L/N(N-1) [39]. Increased network density can indicate that the food web has many generalist species (e.g., omnivores), whereas network density can decrease with highly specialized species having few specific connections. Weighted overall graph clustering coefficient (CL) is a measure of cohesion, it is the”weighted mean of the clustering coefficient of all the nodes each one weighted by its degree” [40]. Average distance (d) is the mean distance between all nodes (where distance is defined as the total number of links between two nodes). Finally, the small-world index (SW) was computed by dividing CL by d. The SW index reflects the network’s resemblance to natural networks [41].

At the node level, three indices were computed: normalized degree centrality (nDC), normalized betweenness centrality (nBC), and trophic level (TL). Largest values of nDC and nBC indices identified the most important (central) nodes. nDC quantifies the number of direct links of a node divided by N-1 [39]. nBC quantifies the centrality on node *i* based on the number of shortest paths between all pairs of nodes *j* and *k* that node *i* is found on [39]. Global network indices, nDC, and nBC were computed in UCINET software [40], and trophic level (TL) using the NetIndices package [42] in R [37]. Trophic level of producers and detritus are set at TL = 1, and for a consumer, TL is calculated as the weighted average of the trophic levels of its food sources plus one [22]. In addition, computed trophic level values for fish and invertebrates were compared to reported values on Fishbase [33] and SeaLifeBase [34] to check for accuracy.

### Energy fluxes

Total biomass data was available for the aggregated networks, the energy fluxes were computed in the respective aggregated networks using the *fluxweb* package in R [43]. For this analysis, four input parameters were used: 1) the binary adjacency matrix based on the aggregated webs, 2) total biomasses (g) for each functional group, 3) average body mass (g) for each functional group, and 4) assimilation efficiencies based on literature (0.906 for animals, 0.545 for plants and cyanobacteria, 0.158 for detritus) [44]. Nodes reported in literature without biomass data (*Aphanius fasciatus*, *Carcinus aestuarii*, Flatfish, Gobidae, *Maja squinado*, *Upogebia pusilla*) were omitted from the energy fluxes analysis. We note here that average body size or biomass data for adult organisms is only a rough approximation in the case when larvae and juveniles play an important role in ecosystem dynamics.

Because the energy fluxes were estimated from top-down, organisms at the basal trophic level (i.e., producers, cynobacteria, detritus) can have any numeric value (we put 1 for their biomasss in the model) and their metabolic rate was set to zero [43]. Energy fluxes were computed using the *fluxing* function with the above four parameters and bioms.prefs = TRUE (meaning that feeding preferences take into account prey biomass data) and ef.level =“prey” (meaning that feeding efficiencies are based on the prey, because most groups feed on a variety of resource nodes, as detailed in reference [43]). The numerical output of fluxweb was exported as a table (S3 Table), log-transformed (to help visual interpretation) and displayed as weighted interaction matrices.

## Results

The environmental characteristics and primary production (measured by chlorophyll-*a*) changed considerably over time (Table 1). Mean annual chlorophyll-*a* increased from less than 1 μg/L in the PRE to 54 μg/L in the POST, meaning that the system shifted from an oligotrophic system to an eutrophic system. Mean annual water temperature also increased considerably, from 15.33°C to 19.28°C. Mean water depth increased by 10 cm. The other variables (summer water temperature, mean annual water salinity, and mean summer water salinity) also increased, but less drastically (Table 1).

Global metrics of the pre- and the post-transformation aggregated food webs showed similar values (Table 3). In order to characterize both food webs, we ran network analysis on both the raw (compiled from literature) and the aggregated networks. Based on the raw networks, we found that there was slightly more information available on the POST network (N = 56, L = 119) than on the PRE network (N = 41, L = 100) (S2 Table). In the PRE network, the nodes having the top five largest nDC values (i.e., nodes with the greatest number of direct links) were: *Atherina boyeri* and Detritus (nDC = 0.35), Crustacea (nDC = 0.28), Gastropoda (nDC = 0.25), *Anguilla anguilla* (juveniles), Polychaeta (sedentary), *Ruppia cirrhosa*, and *Lamprothamnium papulosum* (nDC = 0.23), and Bivalvia and Polychaeta (mobile) (nDC = 0.20). Considering nBC, the nodes with largest values (i.e., the most central nodes) in the PRE network were: Detritus (nBC = 36.93), *Atherina boyeri* (nBC = 17.48), Crustacea (nBC = 14.49), Gastropoda (nBC = 13.63), and Bivalvia (nBC = 7.75; S4 Table). This means that these nodes act as bridges within the network and would have a large impact if removed. Thus, based on both centrality indices (i.e., nDC and nBC), *Atherina boyeri*, Crustacea, Gastropoda, and Detritus are the most important nodes in the PRE network. In the POST network, the most central nodes for nDC were Detritus (nDC = 0.40), Nanocyanobacteria and Picocyanobacteria (nDC = 0.38), *Engraulis encrasicolus* (nDC = 0.22), Copepoda and Polychaeta (nDC = 0.15), Crustacea, Diatoms and Shrimp (nDC = 0.13). For nBC, *Engraulis encrasicolus* (nBC = 29.95), Detritus (nBC = 24.85), Picocyanobacteria and Nanocyanobacteria (nBC = 18.20), Copepoda (nBC = 16.89) and Crustacea (nBC = 12.50) had the largest values (S4 Table). Thus, considering both nDC and nBC indices, the most important nodes were *Engraulis encrasicolus*, Detritus, Picocyanobacteria, Nanocyanobacteria, and Copepoda in the POST network.

**Table 3:** Global metrics of the pre-transformation (PRE) and post-transformation (POST) aggregated networks.

Global metrics (abbreviation)	PRE	POST
Number of nodes (N)	23	23
Number of links (L)	55	52
Network density (D)	0.217	0.221
Weighted overall graph clustering coefficient (CL)	0.174	0.142
Average distance (d)	2.146	2.217
Small world index (CL/d)	0.081	0.064

**Fig 2:**
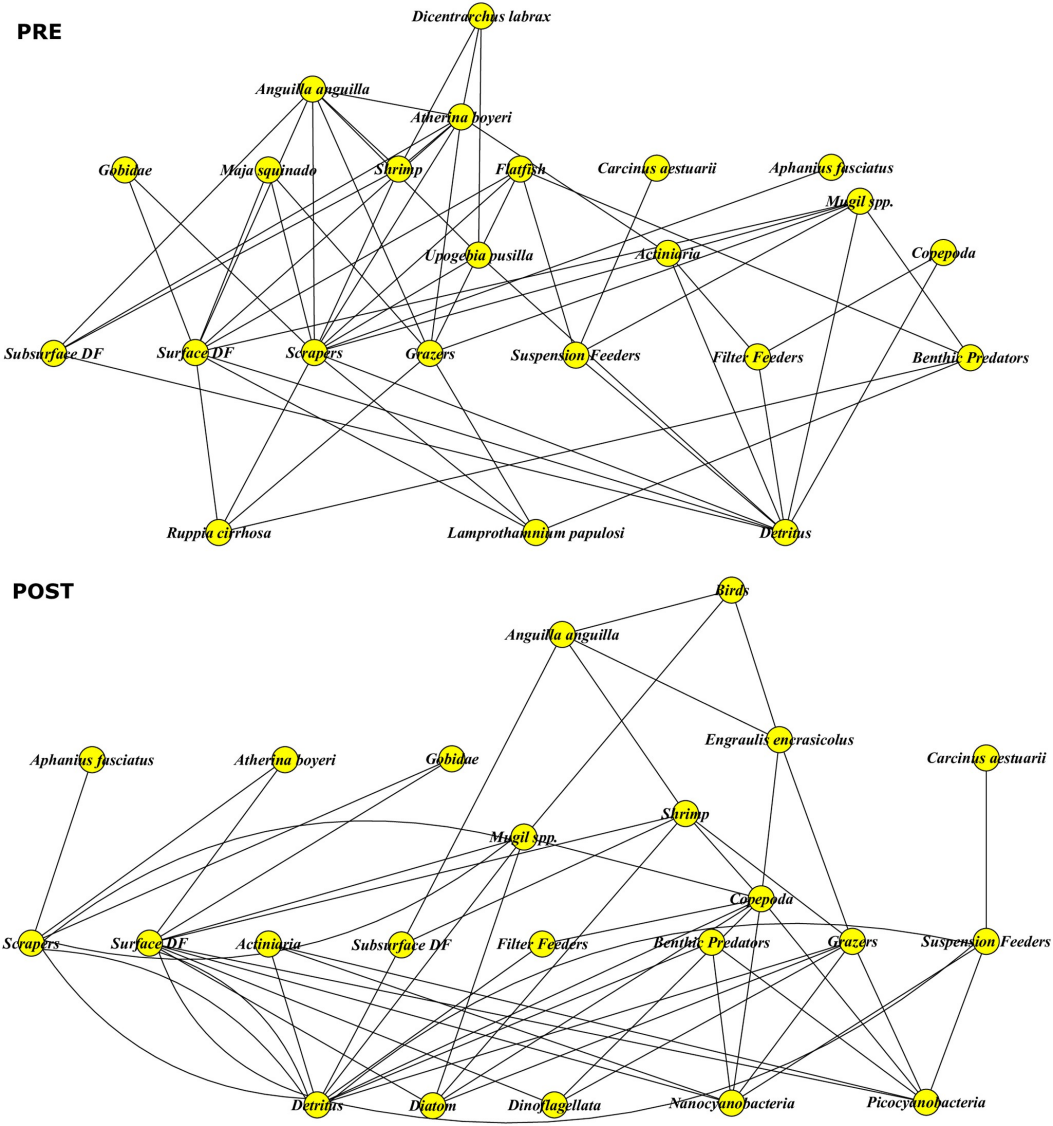
Food webs of the functional groups in the aggregated pre-transformation (PRE) and post-transformation (POST) networks (DF = Deposit Feeders).

**Fig 3:**
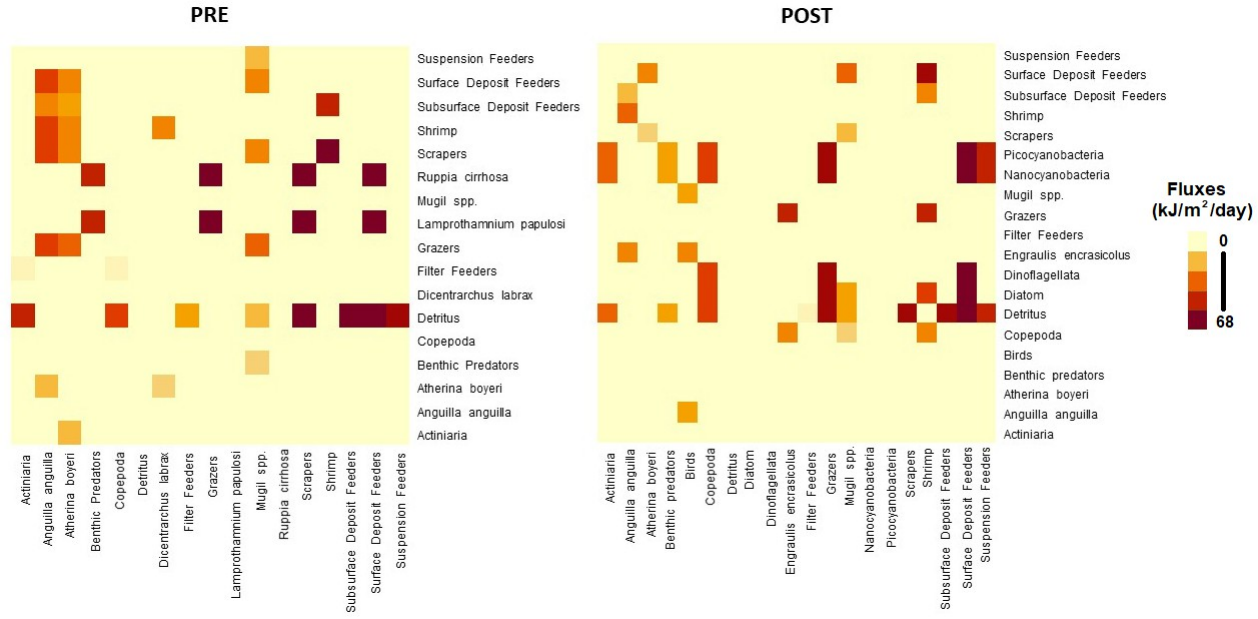
Trophic flux matrices of food webs of the pre (PRE) and post-transformation (POST) networks arranged by trophic level. Units are in base-10 log scale.

Trophic level and energy fluxes were computed on the aggregated networks (S3 Table) and visualized in Figs 2 and 3. For fish species, all computed trophic level values matched the reported ranges in both networks, except for *Mugil* spp. complex in the PRE (3.00 computed vs 2.65 +/- 0.25 on FishBase). Small differences can come from local diet differences in Comacchio vs FishBase reports. The *Mugil* spp. is a combination of four mullet species with feeding on Gastropoda and Bivalves in Comacchio. The maximun fish TL decreased from 3.92 (*Dicentrarchus labrax*) in the PRE to 3.64 (*Anguilla anguilla*) in the POST. The two networks share 16 aggregate nodes: five groups had a decrease in TL (*Atherina boyeri* (3.25 to 3.00), Shrimp (3.00 to 2.83), *Mugil* spp. (2.88 to 2.69), Copepoda (2.50 to 2.17), Actiniaria (2.50 to 2.00)), or no change (*n* = 10), and *Anguilla anguilla* had slight increase in TL (3.39 to 3.64), but within the accepted range of variance (+/- 0.3 S.E.) (Fig 2, S5 Table).

There was considerable change in trophic fluxes between the pre- and post-transformation periods (Fig 3). In the PRE, seagrasses (*Ruppia cirrhosa* and *Lamprothamnium papulosi*) and detritus had the largest fluxes. In the POST, seagrasses disappeared and phytoplankton (e.g., diatom, dinoflagellata), cyanobacteria and detritus were the base of the food web. In particular, Detritus showed more intense fluxes in the POST than in the PRE. The importance of Copepoda in the trophic energy transfer increased in the POST. Shrimp were important in both PRE and POST systems, having fewer but more intense fluxes in the PRE, and multiple but weaker fluxes in the POST. Fish (*Atherina boyeri* and *Anguilla anguilla*) were more important pathways in the PRE than in the POST. The energy flow of *Mugil* spp. complex does not show difference between the two periods. In the POST, a new energy pathway was created by *Birds* (this was not established in the lagoon in the PRE version).

## Discussion

### Temporal comparison

In order to have a meaningful comparison, the pre- and post-transformation networks were aggregated into functional groups (resulting in food webs with similar network topologies). Differences inferred from these comparable aggregated networks thus can be interpreted in an ecological sense. For the first time, we compiled and analysed food webs and energy fluxes, using the most complete information available on all aquatic taxa.

The trophic network describing the aquatic ecosystem in the Comacchio Lagoon changed as a result of the eutrophication event in the early 80s. The larger number of fluxes in the POST network, that are of weaker strength, indicates a possibly more resistant ecosystem with less efficient energy transfers, in terms of the trophic structure [45]. Most differences found confirm previous studies on the lagoon [31, 32].

### Changes in producers

In the pre-trasformation period, the Comacchio Lagoon was characterized by seagrass meadows of *R*. *cirrhosa* and *L*. *papulosum* [46], which resulted in the largest trophic flux. After the eutrophication event, the water turbidity increased, and seagrasses were progressively lost and replaced by cyanobacteria and phytoplankton as described by Sorokin [31, 32]. Given the crucial role of seagrasses in providing nursery grounds, habitat, shelter, and grazing areas for a multitude of fish and invertebrates and in maintaining food webs [47], the loss of seagrasses in aquatic ecosystems, and also in Comacchio Lagoon, has a significant impact on food webs, biodiversity and ecosystem services [48]. This drastic change was also confirmed by the 54-fold increase in Chl-*a*, as an indicator of phytoplankton, in the post-trasformation period [49]. Elevated concentrations of Chl-*a* remained high in the lagoon in recent monitoring sampling (*Lanzoni*, *unpublished data*).

A similar event was reported in the Mar Menor Lagoon in Spain, which experienced a prolonged eutrophication process due to increased nutrient inputs. This culminated in an ecological crisis in 2016, marked by a significant decrease in water transparency and widespread seagrass die-off [50].

### Changes in consumers

The loss of seagrasses meadows led to the decline of large zooplankton filter feeders (such as planktonic invertebrate larvae) and large phytal macroinvertebrates as *Idotea balthica* and led to the increase of the importance of Copepoda in the post-transformation, quantified by both centrality indices (nDC and nBC) and by the increase in trophic fluxes. Copepoda can have a variable trophic level in different ecosystems, based on which level its prey is consumed from [51, 52]. For example, in the North Atlantic, Copepoda are positioned around the third trophic level, feeding on ciliates [52, 53]. Wherease in Peru, abundant upwelling nutrients enable phytoplankton to grow larger and Copepoda are found near the second trophic level feeding directly on phytoplantkon [52, 54]. At Comacchio, the trophic level of Copepoda showed a slight decrease over time. This is an example of food chain shortening, which has been documented in experimental setting (e.g., mesocosms: [55, 56]). As demonstrated in microcosm/mesocosm experiments, nutrient addition allowed diatoms to increase in size and resulted in a trophic switch from copepods consuming phytoplankton directly instead of ciliates [52, 57]. Although we do not have data on ciliates, presumably, trophic switch is the underlying mechanism that happened at Comacchio, being triggered by excessive nutrients via eutrophication. Thus, we can infer, that the Copepoda energy transfer became more important post-transformation. Detritus pathway also increased in the eutrophic system with more energy being transfered to detritivores, as also reported in the Venice Lagoon where increased water turbidity has favored detritivores due to the rise in organic carbon content in the water column [58].

In the Comacchio Lagoon, the change in energy pathways (via increased detritivory and trophic switch of Copepoda) suggests that there is less energy reaching fish with certain species (*Atherina boyeri* and *Anguilla anguilla*) becoming less important in the post-transformation period. In the case of *A*. *anguilla*, as highlighted by Aschonitis et al. [59], the decline in recruitment has been the main contributor to the decline in eels’ abundance in the Comacchio Lagoon, due to global causes such as overfishing of glass eels and their illegal trade outside of the European borders. Furthermore, the few remaining eels have beeb able to adapt to changes in the ecosystem. Eels up to 28.9 cm in size selectively prey on *Palaemon* shrimp and then move on to the new resource that has become available in the same size range of the *A*. *boyeri*, i.e. the anchovy *Engraulis engrasicolus* [60]. This finding is also confirmed from network centrality (nDC) which found the *Atherina boyeri* as the most central node in the pre-transformation system, whereas *Engraulis encrasicolus* was the most important in the post-transformation period. This important substitution in the two trophic networks is due to the major change in primary producers following the eutrophication crisis of the early 1980s, which led to the disappearance of macrophytic vegetation and its replacement by phytoplankton. *A*. *boyeri* is typical of transitional ecosystems with clear waters dominated by the presence of macrophytic vegetation, where it selectively preys on phytobenthos [33]. In contrast, *E*. *encrasicolus* is a typical pelagic species that only occasionally, mainly in spring, visits the transitional waters of the Comacchio area in the Adriatic [61, 62]. As a result of the ecosystem change in the Comacchio Lagoon, which has led to a high availability of phytoplankton and herbivorous zooplankton, this typically planktophagous species has progressively increased in the Comacchio Lagoon until it has become the main node in the energy transfer of the ecosystem in the post-transformation period.

The trophic level of fish decreased over time, consistent with the overall shortening of the food chain. In addition, the identity of the top fish species changed from *Dicentrarchus labrax* in the seagrass habitat to *Anguilla anguilla* in the eutrophic system. These results could be caused by the disappearance of aquatic vegetation (e.g. *Ruppia* spp.) and the associated increase in water turbidity along a trophic cascade of effects, from the disappearance of large zooplankton filter feeders and large phytal macroinvertebrates to *A*. *boyeri* and *D*. *labrax* [63]. Another aspect to be considered together with the chance of trophic availability is the increase in water turbidity in the post-transformation period, which may affect visual predators such as *D*. *labrax* [64].

The trophic level of *Mugil* spp. complex decreased over time, because the mullet species switched from consuming mainly gastropods and bivalvia in the pre-transformation to crustacea and polychaetes post-transformation. Grazer (gastropod) biomass decreased about six-fold in the eutrophic system probably reflecting the negative trend of seagrasess which can act as sink of grazers [65]. The energy flow, however, of *Mugil* spp. complex does not show difference between the two periods, since they are omnivore species. Omnivore species have the ability to switch resources [66] and they typically exhibit many weak links in the network [67, 68]. Certainly, in Comacchio, this seems to be true, they have many trophic connections, but none of the energy fluxes are very strong. This could also be due to the detail of our data, which does not allow to highlight the prevalence of omnivorous mullet species (such as *Chelon auratus* and *Mugil cephalus*) in the pre- and the prevalence of detritivorous mullet species (*Chelon ramada*) in the post-transformation period (*Lanzoni unpublished data*). Shrimp species were important in both systems, with *Palaemon elegans* having few but more intense fluxes in pre-transformation. After eutrophication, *Crangon crangon* and *Palaemon elegans* had more feeding connections but weaker fluxes.

### Changes in top predators

At the top of the food web, water birds (e.g., Ardeidae) were absent in the pre-transformation period: they were hunted for human consumption and probably also to mitigate their predation on fish, thereby averting economic losses for fishermen. Additionally, the establishment of many bird species in Comacchio did not occur until after the 1980s [65]. In the ‘80s, in the post-transformation period, many bird species (e.g. *Phalacrocorax carbo*) appeared in the North Est of Italy, also favored by the establishment of protected wetland areas [69], and they became part of the food web consuming fish (*Aguilla anguilla*, *Engraulis encrasicolus*, *Mugil* spp.) in Comacchio Lagoon. On the other hand, European protected areas were found to enhance large predators such as birds in food webs as successful outcome of European Directives [70]. In the pre-transformation period, fish were at the top of the food web, probably facilitated by human management of the ecosystem with the goal of maximizing fishing.

### Detritus

Detritus is an important reservoir of nutrients in ecosystem and plays an important role in nutrient cycling and food web dynamics [71]. In Comacchio Lagoon, the detritus resulted important in both pre- and post- transformaiton periods. In particular, the ecosystem switched from macrophyte primary production to cyanobacteria as the primary source of energy and increasingly detritus-based nutrients in the post-transformation period, confirming the previous studies of Sorokin [31, 32]. Despite all efforts to collect accurate data, some data information is still incomplete. For example, data on birds presented are missing species, and there are also knowledge gaps at the base of the food web, with data missing on ciliates, detritus, phytoplankton, and microbial food web components. Filling knowledge gaps for historical period could be challenging as ecological datasets rarely go back more than a few decades [72]. Despite the data gaps, our results are consistent with previous studies that examined only a few taxa [31, 32], suggesting that the missing information was not critical to our findings.

Therefore, environmental monitoring plays a crucial role in preventing data gaps, and in quantifying and incorporating these groups into the food web in future research.

### Climate change greater perspective

In the case of Comacchio Lagoon, the dramatic trasformation of its ecosystem was due to severe human management practices as pointed out by Sorokin. However, it is important to recognize that similar drastic changes triggered by climatic chenge can affect other wetlands in the near future. To put in perspective, climate change is expected to shift aquatic ecosystems toward cyanobacteria dominance, due to the better competitive ability of green-blue algae [73], resulting in major changes in the food web composition and their interactions [74]. Given the climate projections, baseline, long-term, and comparative studies are urgently needed. We need to be able to understand the current (or past) statuses of an area of interest in order to document the changes which gradually (or some more abruptly) occur in these ecosystems. Thus, ecological approaches must step up from single-species studies to ecosystem-level analyses, we must attempt to describe the spatial and temporal patterns and system dynamics. Quantitative approaches are desired, but even qualitative approaches are better than none. Here, we show, that if the ecosystem has already undergone major change (i.e. the ecological status and food web components are drastically different between representative states), a simple approach of connectance food web construction and network analysis can be used to characterize the ecosystem transformation.

## Conclusions

Our study reveals a significant difference in the trophic networks before and after the eutrophication event that occurred in the early 1980s, resulting in some decrease in complexity, increase of flow diversity and an overall shortening of the food chain. A crucial aspect of this change is the disappearance of subaquatic vegetation in the lagoon and the increased importance of cyanobacteria in the post-eutrophication period.

Comparative system-level analyses provide a quantitative, holistic view on changes in ecosystem functioning. Understanding structural changes in patterns and processes is a pre-requisite for assessing changes in ecosystem services and socio-ecological consequences. Managing blue-green infrastructures, fisheries and coastal ecosystems can, thus, benefit from our analysis.

## Supporting information

S1 Table Reference list for the data sources for each variable for the PRE- and POST-transformation periods. (XLS)

S2 Table Predator—Prey edgelist for the raw food webs (as compiled from literature) and for the aggregated food webs (functional groups) for the PRE- and POST-transformation periods. (XLS)

S3 Table Energy fluxes (kJ/m^2^/day) for the PRE-transformation and the POST-transformation aggregated trophic networks. (XLS)

S4 Table Normalized degree centrality (nDC) and normalized betweenness centrality (nBC) values for the raw food webs PRE- and POST-transformation. (XLS)

S5 Table Trophic level (TL) values for the aggregated food webs (*n* = 23 nodes) PRE- and POST-transformation. (XLS)
